# Ultrasound *Versus* Contrast-Enhanced Magnetic Resonance Imaging for Subclinical Synovitis and Tenosynovitis: A Diagnostic Performance Study

**DOI:** 10.6061/clinics/2020/e1500

**Published:** 2020-01-14

**Authors:** Zhongtao Bao, Yanchun Zhao, Shuqiang Chen, Xiaoyu Chen, Xiang Xu, Linglin Wei, Meilian Xiong

**Affiliations:** I Department of Ultrasound, First Affiliated Hospital of Fujian Medical University, Fuzhou, Fujian, 350000, China; II Department of Ultrasound, Fujian Provincial Hospital, Fuzhou, Fujian, 350000, China; III Department of Imaging, First Affiliated Hospital of Fujian Medical University, Fuzhou, Fujian, 350000, China

**Keywords:** Magnetic Resonance Imaging, Metacarpophalangeal Joints, Proximal Interphalangeal Joints, Synovitis, Tenosynovitis, Ultrasound, Wrist

## Abstract

**OBJECTIVES:**

Radiographic manifestations of synovitis (e.g., erosions) can be observed only in the late stage of rheumatoid arthritis. Ultrasound is a noninvasive, cheap, and widely available technique that enables the evaluation of inflammatory changes in the peripheral joint. In the same way, dynamic contrast-enhanced magnetic resonance imaging (MRI) enables qualitative and quantitative measurements. The objectives of the study were to compare the sensitivity and accuracy of ultrasound in detecting subclinical synovitis and tenosynovitis with those of contrast-enhanced MRI.

**METHODS:**

The ultrasonography and contrast-enhanced MRI findings of the wrist, metacarpophalangeal, and proximal interphalangeal joints (n=450) of 75 patients with a history of joint pain and morning stiffness between 6 weeks and 2 years were reviewed. The benefits score was evaluated for each modality.

**RESULTS:**

The ultrasonic findings showed inflammation in 346 (77%) joints, while contrast-enhanced MRI found signs of early rheumatoid arthritis in 372 (83%) joints. The sensitivities of ultrasound and contrast-enhanced MRI were 0.795 and 0.855, respectively, and the accuracies were 0.769 and 0.823, respectively. Contrast-enhanced MRI had a likelihood of 0-0.83 and ultrasound had a likelihood of 0-0.77 for detecting synovitis and tenosynovitis at one time. The two imaging modalities were equally competitive for detecting synovitis and tenosynovitis (*p*=0.055).

**CONCLUSION:**

Ultrasound could be as sensitive and specific as contrast-enhanced MRI for the diagnosis of subclinical synovitis and tenosynovitis.

## INTRODUCTION

Approximately 1% of the Chinese population is affected by rheumatoid arthritis (1). The cause of rheumatoid arthritis, an autoimmune disease, is still unclear, and this condition is considered to be due to both environmental and genetic factors. Rheumatoid arthritis greatly affects the synovium of joints. The symptoms of rheumatoid arthritis are similar to those of many other diseases and conditions, making it difficult to diagnose. If rheumatoid arthritis is not treated, then it results in destruction and disorganization of the joint (2). Chronic disease will lead to conditions such as synovial hypertrophy and angiogenesis (3). Rheumatoid arthritis can be detected using both ultrasound and magnetic resonance imaging (MRI) (4). In addition, both of these modalities have the potential to depict the enhanced permeability of small vessels in the case of angiogenesis.

The pathological conditions observed in early rheumatoid arthritis include thickening of the synovium, bursae, and tendon sheaths. Joint effusion also occurs in the initial stage of rheumatoid arthritis and was found to be associated with bursitis, synovitis, and tenosynovitis. The next stage involves angiogenesis and vascularization of the synovium (5).

Radiographs have been used as traditional methods to diagnose and rule out rheumatoid arthritis. The common features observed using these techniques are marginal erosion, inflammation of the joint tissue, joint subluxation, loss of joint space and osteopenia (6), but such manifestations of rheumatoid arthritis can be observed only in the late stage of the disease. The early radiography findings of rheumatoid arthritis are synovitis and swelling of the soft tissue in the hands. Proliferative synovitis, which is associated with joints of the wrist, hand, ankle, and cervical spine, is responsible for the destruction of bones and cartilage (7).

Diagnostic ultrasound is a noninvasive technique used to image the inside of the body. MRI, a versatile radiology technique, makes use of magnetic fields to pictorially represent anatomical structures and the physiology of the body. Both MRI and ultrasound are highly sensitive techniques and are increasingly being used in clinical practice and research. MRI enables the detection of various conditions, including bone marrow edema. Bone marrow edema is responsible for bone erosion and inflammation. Bone edema is not visible with ultrasound imaging or computed tomography (CT). MRI has the potential to enable visualization in three orthogonal planes and provides details for both the affected bone and the surrounding area of the joints (8).

MRI has the advantages of being able to evaluate peripheral joints in terms of articular and periarticular inflammation, cartilage damage, bone erosion and tendon tearing. Dynamic contrast-enhanced MRI can obtain qualitative, quantitative and semi-quantitative measurements. Additionally, it facilitates the estimation of inflammatory changes and complications of long-standing inflammation and provides a global, instead of local, view of bone, including the bone’s internal structure and periarticular tissue. Ultrasonography is cost-effective. It allows for the evaluation of peripheral joints in cases of inflammation (ulnar nerve stability, luxation, and tendon tearing). Synovial hypertrophy and the degree of joint vascularization can be determined using ultrasonography.

Ultrasound is readily available at various centers, but it is not able to examine the internal structure of bone. Additionally, ultrasound cannot image bone edema, which can be visualized by only MRI. However, contrast-enhanced MRI has requires the administration of contrast agents, has motion artifacts, and has a low resolving power compared to ultrasound (9).

MRI reveals synovitis as a thickening of the synovial membrane. Contrast-enhanced T1 images are more sensitive and accurate in detecting acute synovitis than unenhanced MRI scans. During contrast-enhanced imaging, the enhancement lasts for approximately 5 min after the injection (10). The contrast agent reaches the synovial fluid after 6-10 min, which is when imaging provides the best results.

The objectives of the study were to compare the sensitivities and accuracies of ultrasound and contrast-enhanced MRI in the detection of subclinical synovitis and tenosynovitis in the wrist, metacarpophalangeal joints, and proximal interphalangeal joints.

## MATERIALS AND METHODS

### Ethics approval and consent to participate

The original study protocol (FMU/CL/15/19 dated 21 June 2019) was approved by the Fujian Medical University Review Board. All enrolled patients signed informed consent forms for pathology examinations, radiology examinations, and additional procedures for research purposes and for the publication of the study in all formats, including personal data and images of citable materials, by the publication house irrespective of the time of hospitalization and language of publication. The study adhered to the laws of China, the 2008 Helsinki Declaration, and the Strengthening the Reporting of Observational Studies in Epidemiology (STROBE): cross-sectional statement.

### Inclusion criteria

Patients aged 18 years or older with a history of joint pain and morning stiffness between 6 weeks and 2 years were included in the study.

### Exclusion criteria

Patients who had confirmed rheumatoid arthritis were excluded from the study. Patients who had not undergone all imaging methods were also excluded from the analysis. Pregnant females were excluded from the analysis.

### Clinical data collection

Data regarding the demographic characteristics and laboratory test results of patients were collected from the medical records of the institute.

### Ultrasound assessment

The wrists, proximal interphalangeal joints, and metacarpophalangeal joints were examined using an ultrasound instrument (Acuson Sequoia, Siemens Healthineers, Erlangen, Germany) that utilizes a15 MHz linear array probe (Siemens Healthineers, Erlangen, Germany). The examination was performed with the subject sitting in an upright posture, and the fully pronated hand was positioned on a cushion (11). The scan plane was marked on the skin with a marker. In case of the wrist, scanning of the dorsal site was performed. Scanning was performed in a transverse plane from the superior to the inferior site. Scanning of finger joints was performed in the longitudinal plane only. Scanning of the first metacarpophalangeal joint and all proximal interphalangeal joints was performed from the ulnar to the radial side in a 180° arc. The scanning of the second and the fifth metacarpophalangeal joints was performed from the dorsal side in a 150° arc, while that of the third and the fourth joint were performed in a 120° arc (Table 1). The color Doppler ultrasound setting parameters were as follows: 7 MHz Doppler frequency and Nyquist limit of 0.014 m/s. The scanning process with the color Doppler apparatus took an average of 15 min for each patient. Sonographers at ach institute (minimum 3 years of experience) were involved in the ultrasonographic assessments.

### Contrast-enhanced magnetic resonance imaging

The wrists, proximal interphalangeal joints, and metacarpophalangeal joints were examined by 3.0 Tesla MRI (Siemens Healthineers, Erlangen, Germany). The patients were instructed to stay in a motionless position by placing their hands on their heads. The patients were injected with gadodiamide (Xian Wanlong Pharmaceutical Co. Ltd, Beijing, China) at a concentration of 0.05 mM/kg of body weight (12). The imaging parameters were as follows: 25-26 ms repetition time, 50° flip angle, 0 mm gap, 1 mm slice thickness, and 6-8 min examination time. Radiologists at each institute (minimum 3 years of experience) were involved in the MRI scans.

### Image analysis

If color was observed on the ultrasound scan, then it the joint(s) were considered to have activity, and if no color was found on the ultrasound scan, then the joint(s) were considered to have no activity. In the same manner, if contrast enhancement was found on MR images, then the joint(s) were considered to have activity; otherwise, the joints were not considered to have activity.

### Benefits score analysis

The benefits score was evaluated for each modality as per Eq. 1 and 2 (13):


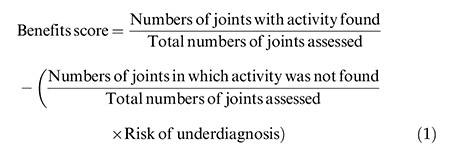



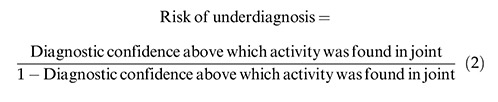


### Statistical analysis

The independent chi-square test was applied for the analysis to determine whether contrast-enhanced MRI and ultrasound were equally effective in detecting the imaging manifestations of early rheumatoid arthritis at 95% of the level of significance (2).

## RESULTS

### Enrollment

**Figure 1:**
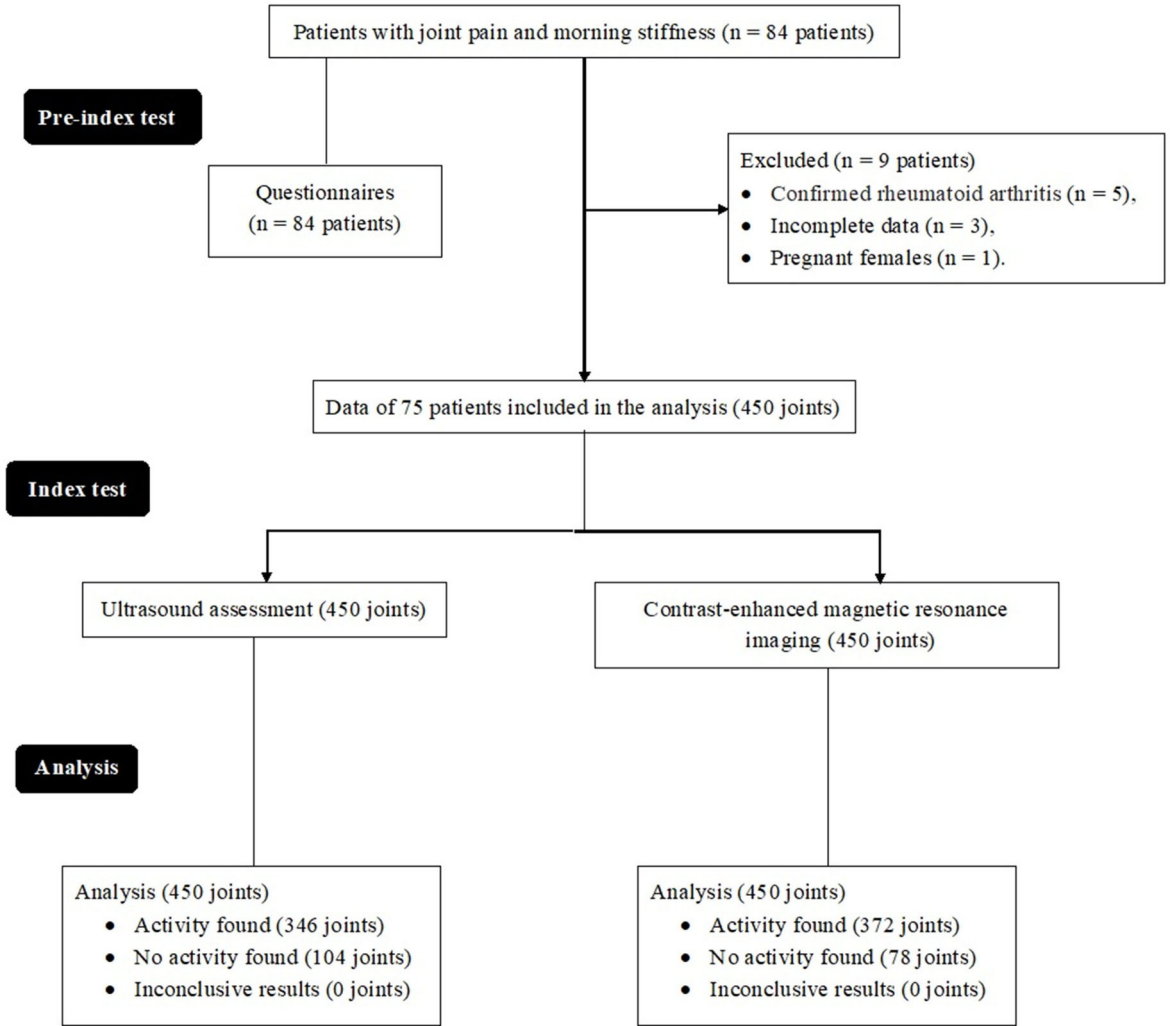
Flow diagram of the analysis.

From 2 January 2019 to 1 April 2019, a total of 84 patients with joint pain and morning stiffness (symptoms duration between 6 weeks and 2 years) were recruited at the Department of Rheumatology of the First Affiliated Hospital of Fujian Medical University, Fuzhou, Fujian, China and the Fujian Provincial Hospital, Fuzhou, Fujian, China. Among them, five patients had confirmed rheumatoid arthritis (positive rheumatoid factor test), the complete data of three patients were not available as institutional records, and one female was pregnant. Therefore, these patients were excluded from the analysis. The demographic, clinical, and radiological data of 75 patients were collected and analyzed (Figure 1).

### Demographic and clinical characteristics

Among the enrolled patients, 77% were female. All enrolled patients had normal erythrocyte sedimentation rates (normal values: 0-22 mm/h for males and 0-29 mm/h for females) and normal rheumatoid factor tests. All patients had moderate body mass index and hypertension along with worsened morning stiffness, which may be caused by synovitis or tenosynovitis. The other demographic and clinical characteristics of the enrolled patients are reported in Table 2.

### Radiological examinations

**Figure 2:**
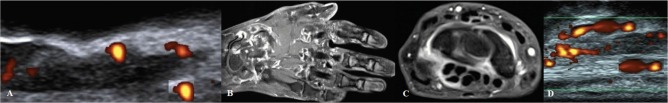
**A.** Ultrasound image (longitudinal view) of a female patient (age 48 years) with inflammatory joint pain showing synovitis in the first proximal interphalangeal joint. **B.** Contrast-enhanced magnetic resonance image of the hand of a male patient (age 49 years) with inflammatory joint pain showing inflammatory cysts, erosion, and synovitis in radiocarpal, carpometacarpal, midcarpal, and metacarpophalangeal joints. **C.** T1-weighted contrast-enhanced magnetic resonance image of the carpalia of a female patient (age 51 years) with inflammatory joint pain showing synovitis in the joints. **D.** Ultrasound image (longitudinal view) of the extensor carpi ulnaris tendon of a female patient (age 47 years) with inflammatory joint pain showing tenosynovitis.

Ultrasound revealed a thick synovial sheath with or without hyperemia (Figure 2A). On the contrast-enhanced MR images, the inflamed synovium showed fast enhancement and lasted for 5 min after the administration of the contrast agent (Figure 2B). The contrast-enhanced MR images clearly defined the tendon sheath and inflammation in the tendon sheath (Figure 2C). Ultrasound revealed hypoechoic and abnormally thickened intra-articular tissue (Figure 2D).

### Agreement between the two imaging modalities

Color Doppler ultrasound revealed inflammatory activity and thickening of the synovial sheath in 346 (77%) out of 450 joints, while MRI revealed signs of early rheumatoid arthritis in 372 (83%) out of 450 joints (Table 3).

**Figure 3:**
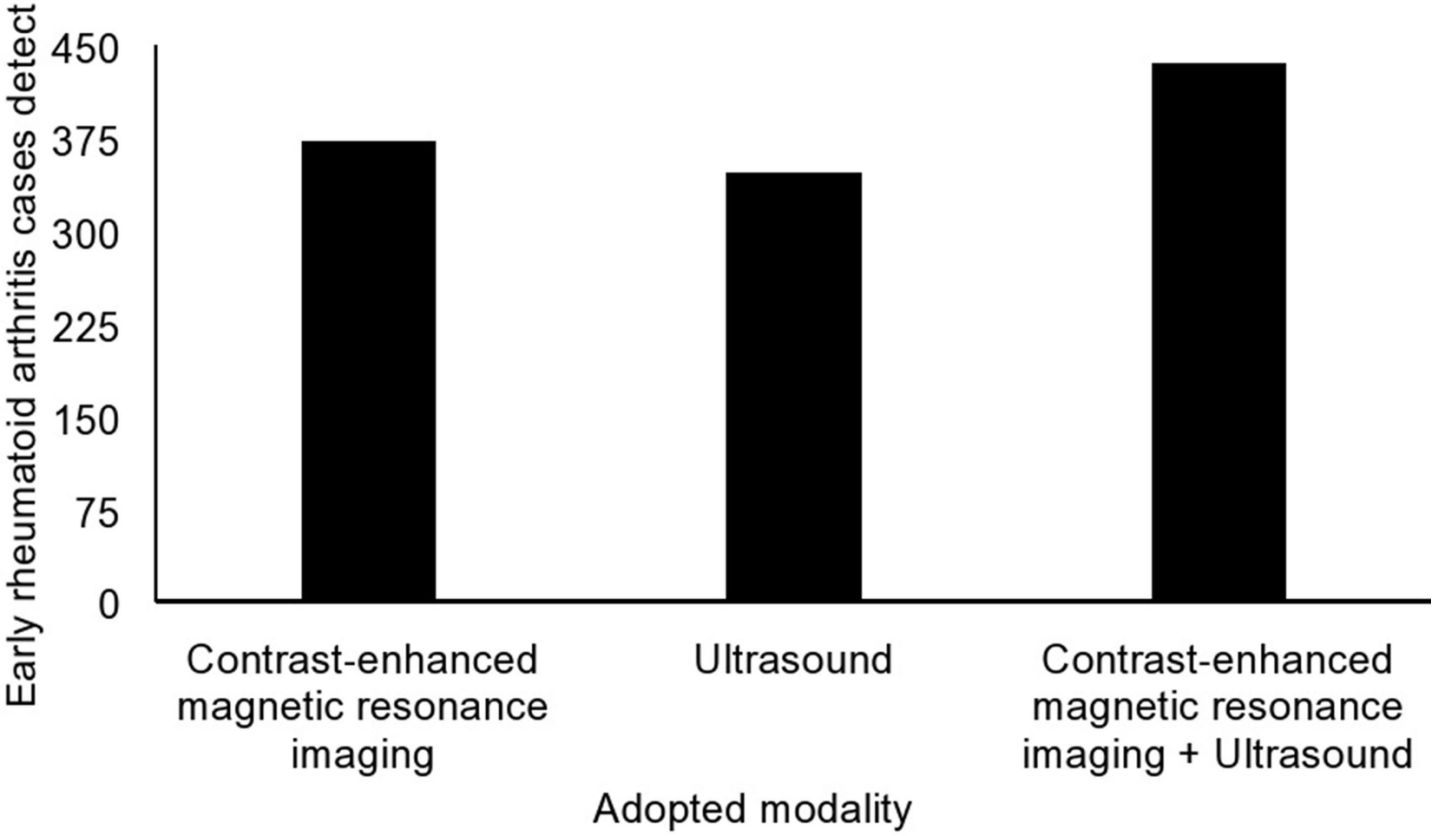
Detection of rheumatoid arthritis using different modalities.

Ultrasound and contrast-enhanced MRI had the sensitivities of 0.795 and 0.855 and accuracies of 0.769 and 0.823, respectively, for detecting inflammatory activity and thickening of the synovial sheath (Figure 3).

According to the statistical analysis, the two imaging modalities were equally competitive for detecting synovitis and tenosynovitis (*p*=0.055), and both techniques can be used according to the diagnostic needs and availability.

### Benefits score analysis

**Figure 4:**
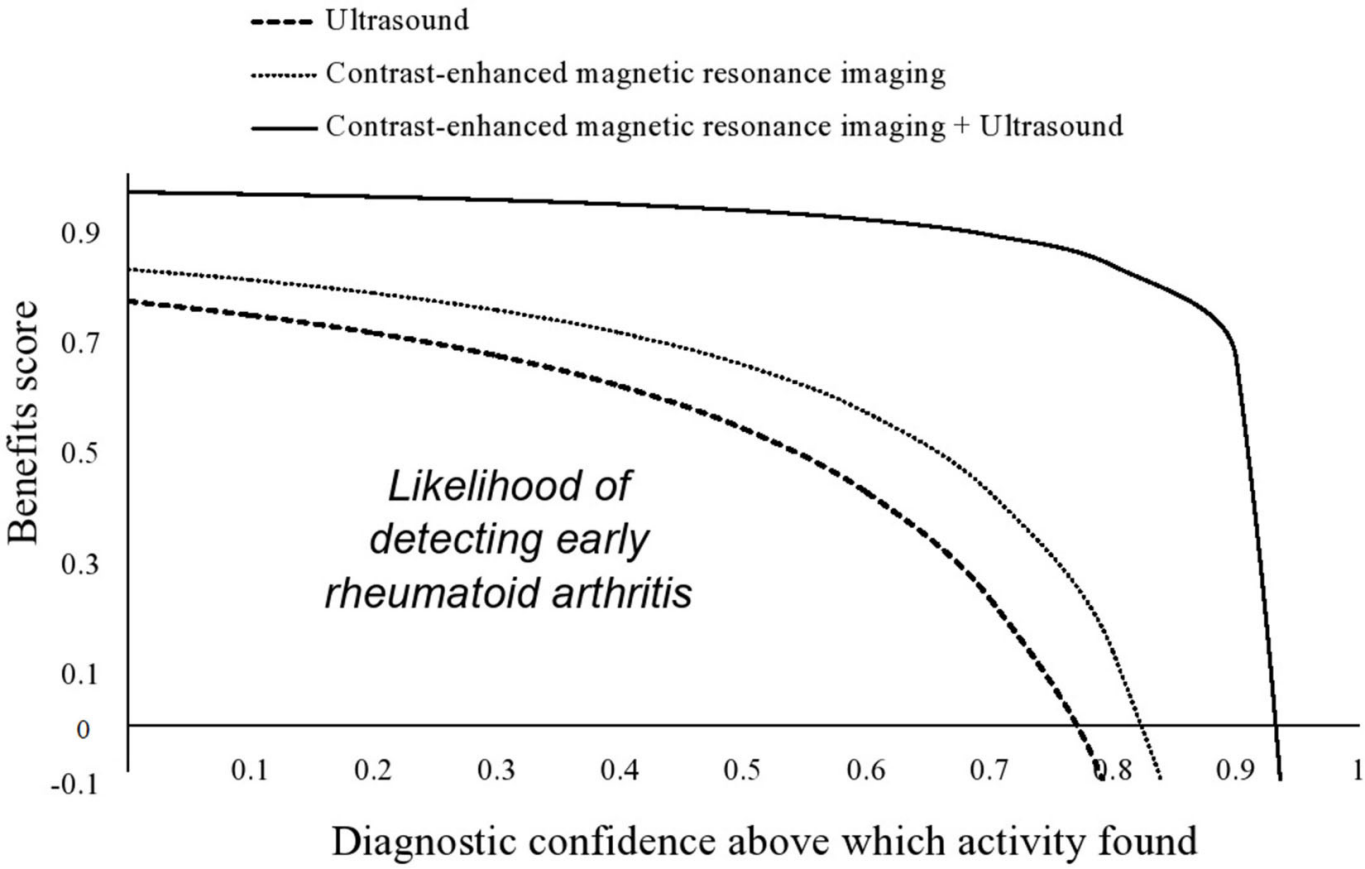
Benefits score analysis.

Contrast-enhanced MRI (0–0.83) had a better likelihood of detecting synovitis and tenosynovitis than ultrasound (0–0.77). When the score was 0.83, contrast-enhanced MRI had a risk for underdiagnosing patients, and when the score was above 0.77, ultrasound had the risk of underdiagnosing patients (Figure 4).

There were no adverse or unwanted effects observed during the study.

## DISCUSSION

This study aimed to compare two imaging modalities, contrast-enhanced MRI and ultrasound, for their abilities to detect inflammatory characteristics of rheumatoid arthritis, including synovitis and tenosynovitis. A total of 75 patients were included in the study, and 450 joints were examined. This study showed that ultrasonography was as effective, sensitive, and accurate as contrast-enhanced MRI (*p*=0.055). The results of the study were in line with those of retrospective studies (2,14). Contrast-enhanced MRI is a tedious and expensive diagnostic technique for the detection of synovitis and tenosynovitis (2,15). Furthermore, the inter- and intraobserver reliabilities of ultrasound were comparable to those of MRI (16). Ultrasound is a good substitute for contrast-enhanced MRI for the diagnosis of subclinical synovitis and tenosynovitis.

The study enrolled patients who were symptomatic for between 6 weeks and 2 years. The present classification criteria for rheumatoid arthritis are based on the erosion and the absence of other indicators (17). Until 2002, patients with a disease duration shorter than 2 years were classified as having early rheumatoid arthritis. Currently, the latest definitions for early rheumatoid arthritis are a disease duration of 12 months from symptom onset and that for very early rheumatoid arthritis is 3 months from symptom onset (18). Early treatment of undifferentiated arthritis and very early rheumatoid arthritis seems to represent a window of opportunity in terms of improving clinical and pharmacoeconomic outcomes. Thus, a reliable imaging technique is essential to confirm clinical findings.

There were 89 joints that were not imaged by ultrasound but were characterized as having some sort of inflammatory reaction and requiring contrast-enhanced MRI. The cases of bone marrow edema were left undetected by ultrasound. Bone marrow edema can only be detected by MRI (19) due to the spatial resolution of ultrasound (2). The results of the study were in line with those of a longitudinal follow-up study (15). Ultrasound is a reliable, cheap, highly available, and widely accepted tool for the detection of subclinical synovitis and tenosynovitis.

The study reported that ultrasound had a sensitivity of 0.795 and contrast-enhanced MRI had a sensitivity of 0.855 for detecting synovitis and tenosynovitis. The results of the study were consistent with the results of available retrospective studies (20,21). In a retrospective study, the sensitivity of conventional radiography in the early detection of bone erosion was just 0.13, while that of MRI was 0.98 and that of ultrasound was observed to be 0.63 (15). The high accuracy and sensitivity are why it has become a trend to diagnose early rheumatoid arthritis using MRI (22). Ultrasound or sonography is also a reliable technique for the detection of bone erosion, especially in cases of early rheumatoid arthritis, compared to other conventional techniques.

Ultrasound was found to have a lower likelihood of detecting early rheumatoid arthritis than contrast-enhanced MRI. The results of the study were in line with those of a retrospective study (2). In some patients, tenosynovitis is the only finding of rheumatoid arthritis. Any tendon can be affected by tenosynovitis. At the level of the wrist, any extensor tendon sheath from compartments I to VI can be widely involved, unlike the flexors. To observe tenosynovitis, the recommended sequence for MRI is T1-weighted imaging. Ultrasound reveals a thick synovial sheath with or without hyperemia (23). Excess fluid within the tendon sheath can also be observed (24). Ultrasound cannot completely replace contrast-enhanced MRI in detecting inflammation of the limb joints and their soft tissues.

There are a few limitations for both techniques; for example, bone marrow edema, an early sign of rheumatoid arthritis, developed in some joints but was not detectable by ultrasonography. MRI does this task well, but requires injections of contrast agents; in some patients, the use of contrast agents is not permitted, according to guidelines published in the European Journal of Ultrasound (25).

An example of the limitations of this study is that the randomized trial had better outcomes than the medical records review study. The demographic and clinical conditions also have effects on tenosynovitis and synovitis, but the study did not evaluate the confounding effects of such parameters on the outcomes of the imaging methods. The MRIs were examined by one radiologist only.

## CONCLUSION

This study provides new evidence to support that ultrasound has a similar sensitivity and accuracy for the diagnosis of subclinical synovitis and tenosynovitis as contrast-enhanced MRI. Patients who experience joint pain and morning stiffness should undergo sonographic assessments to detect early signs of inflammation and/or structural damage and to start the appropriate treatment to prevent bone and cartilage destruction.

## AUTHOR CONTRIBUTIONS

All authors read and approved the final version of the manuscript manuscript for publication. Bao Z and Zhao Y contributed equally to data curation and literature review of the study and draft, review, and edited the manuscript for intellectual content. Chen S was the project administrator, contributed to data curation, resources, and literature review of the study. Chen X contributed to the investigation, validation, resources, and literature review of the study. Xu X contributed to software, formal analysis, validation, and literature review of the study. Wei L contributed to data curation, software, resources, and literature review of the study. Xiong M contributed to funding, formal analysis, data curation, and literature review of the study. All authors agree to be accountable for all aspects of work ensuring integrity and accuracy.

## Figures and Tables

**Table 1:** Different sites of the joints analyzed using color Doppler ultrasonography.

Joint	Sites analyzed
Wrist	Ulnar carpal recesses
	Inter carpal recesses
	Radio carpal recesses
	Volar carpal recesses
Proximal interphalangeal and metacarpophalangeal joints	Lateral recesses
	Volar carpal recesses
	Dorsal recesses

**Table 2:** Anthropologic, social, demographic and clinical characteristics of the enrolled patients.

Parameters		Mean value
Patients included in the analysis		75
Sex	Male	17 (23)
	Female	58 (77)
Mean age (years)		51.2±9.8
BMI (body mass index; kg/m^2^)	Male	24.4
	Female	25.7
Mean systolic blood pressure (mmHg)		127±12.3
Mean diastolic pressure (mmHg)		73±11.7
Duration of morning stiffness (min)		42±19
Mean duration of disease (months)		9.10±6.4
*Erythrocyte sedimentation rate (mm/h)	Male	18.12±2.45
	Female	25.24±3.42
Time working on a laptop/day	Does not work on laptop	41 (55)
	Less than 3 h	21 (28)
	3 to 7 h	9 (12)
	More than 7 h	4 (5)
Daily cycling in morning or evening	Yes	14 (19)
	No	61 (81)
DAS28 score		4.01±0.99

Categorical data are presented as numbers (percentages), and continuous data are presented as the mean ± SD.

DAS28 score: Disease activity score of 28 joints.

* Normal value: 0-22 mm/h for males and 0-29 mm/h for females.

**Table 3:** Agreement between the two imaging modalities used.

	No activity found using ultrasound	Activity found using ultrasound	Total
Total no. of joints reviewed	450	450	450
No activity found via magnetic resonance imaging	15 (3)	63 (14)	78 (16)
Activity found via magnetic resonance imaging	89 (20)	283 (63)	372 (83)
Total no. of joints	104 (23)	346 (77)	450 (100)

Data are represented as a number (percentage).

## References

[1] Xiang YJ, Dai SM (2009). Prevalence of rheumatic diseases and disability in China. Rheumatol Int.

[2] Xu H, Zhang Y, Zhang H, Wang C, Mao P (2017). Comparison of the clinical effectiveness of US grading scoring system vs MRI in the diagnosis of early rheumatoid arthritis (RA). J Orthop Surg Res.

[3] Turan A, Celtikci P, Tufan A, Ozturk MA (2017). Basic radiological assessment of synovial diseases: a pictorial essay. Eur J Rheumatol.

[4] Sudol-Szopinska I, Jurik AG, Eshed I, Lennart J, Grainger A, Ostergaard M (2015). Recommendations of the ESSR Arthritis Subcommittee for the Use of Magnetic Resonance Imaging in Musculoskeletal Rheumatic Diseases. Semin Musculoskelet Radiol.

[5] Hodgson RJ, O'Connor P, Moots R (2008). MRI of rheumatoid arthritis image quantitation for the assessment of disease activity, progression and response to therapy. Rheumatology.

[6] Villeneuve E, Emery P (2009). Rheumatoid arthritis: what has changed?. Skeletal Radiol.

[7] Boutry N, Morel M, Flipo RM, Demondion X, Cotten A (2007). Early rheumatoid arthritis: a review of MRI and sonographic findings. AJR Am J Roentgenol.

[8] Freeston JE, Bird P, Conaghan PG (2009). The role of MRI in rheumatoid arthritis: research and clinical issues. Curr Opin Rheumatol.

[9] Rowbotham EL, Grainger AJ (2011). Rheumatoid arthritis: ultrasound versus MRI. AJR Am J Roentgenol.

[10] Narváez JA, Narváez J, De Lama E, De Albert M (2010). MR imaging of early rheumatoid arthritis. Radiographics.

[11] Hamdi W, Miladi S, Matallah K, Bouaziz M, Kaffel D, Zouch I (2017). AB0278 Ultrasound examination in diagnosis of early rheumatoid arthritis. Ann Rheum Dis.

[12] Boyesen P, Haavardsholm EA, Ostergaard M, van der Heijde D, Sesseng S, Kvien TK (2011). MRI in early rheumatoid arthritis: Synovitis and bone marrow oedema are independent predictors of subsequent radiographic progression. Ann Rheum Dis.

[13] Fitzgerald M, Saville BR, Lewis RJ (2015). Decision curve analysis. JAMA.

[14] Halil H, Tekeoglu I, Sag MS, Harman S (2015). Diagnostic value of musculoskeletal ultrasound in newly diagnosed rheumatoid arthritis patients. Turk J Phys Med Rehab.

[15] Rahmani M, Chegini H, Najafizadeh SR, Azimi M, Habibollahi P, Shakiba M (2010). Detection of bone erosion in early rheumatoid arthritis: ultrasonography and conventional radiography versus non-contrast magnetic resonance imaging. Clin Rheumatol.

[16] Baillet A, Gaujoux-Viala C, Mouterde G, Pham T, Tebib J, Saraux A (2011). Comparison of the efficacy of sonography, magnetic resonance imaging and conventional radiography for the detection of bone erosions in rheumatoid arthritis patients: a systematic review and meta-analysis. Rheumatology.

[17] Aletaha D, Neogi T, Silman AJ, Funovits J, Felson DT, Bingham CO 3rd (2010). 2010 Rheumatoid arthritis classification criteria: an American College of Rheumatology/European League Against Rheumatism collaborative initiative. Arthritis Rheum.

[18] Espinoza F, Fabre S, Pers YM (2016). Remission-induction therapies for early rheumatoid arthritis: evidence to date and clinical implications. Ther Adv Musculoskelet Dis.

[19] Rios AM, Rosenberg ZS, Bencardino JT, Rodrigo SP, Theran SG (2011). Bone marrow edema patterns in the ankle and hindfoot: distinguishing MRI features. AJR Am J Roentgenol.

[20] Ogishima H, Tsuboi H, Umeda N, Horikoshi M, Kondo Y, Sugihara M (2014). Analysis of subclinical synovitis detected by ultrasonography and low-field magnetic resonance imaging in patients with rheumatoid arthritis. Mod Rheumatol.

[21] Fukuba E, Yoshizako T, Kitagaki H, Murakawa Y, Kondo M, Uchida N (2013). Power Doppler ultrasonography for assessment of rheumatoid synovitis: comparison with dynamic magnetic resonance imaging. Clin Imaging.

[22] Heidari B (2011). Rheumatoid Arthritis: Early diagnosis and treatment outcomes. Caspian J Intern Med.

[23] Sudol-Szopinska I, Jans L, Teh J (2017). Rheumatoid arthritis: what do MRI and ultrasound show. J Ultrason.

[24] Hassan R, Hussain S, Bacha R, Gillani SA, Malik SS (2019). Reliability of Ultrasound for the Detection of Rheumatoid Arthritis. J Med Ultrasound.

[25] Piscaglia F, Nolsoe C, Dietrich CF, Cosgrove DO, Gilja OH, Bachmann Nielsen M (2012). The EFSUMB Guidelines and Recommendations on the Clinical Practice of Contrast Enhanced Ultrasound (CEUS): update 2011 on non-hepatic applications. Ultraschall Med.

